# Genetic evolution analysis of AKAV Gc gene

**DOI:** 10.3389/fvets.2025.1715626

**Published:** 2025-11-13

**Authors:** Xiaolin Lan, Qipeng Zhang, Ruqing Zeng, Fang Liang, Jiaman Li, Gan Li, Feng Li, Yaqiong Ye, Mengmeng Zhao

**Affiliations:** 1 Guangdong Provincial Key Laboratory of Animal Molecular Design and Precise Breeding, School of Animal Science and Technology, Foshan University, Foshan, Guangdong, China; 2 Center of Animal Epidemic Disease Prevention Control, Chongzuo, Guangxi, China

**Keywords:** Akabane disease, Akabane virus, Gc gene, Gc gene genetic variation, phylogeny

## 1 Introduction

Akabane disease (AKAD), also known as Akabane encephalomyelitis, is an arthropod-borne infectious disease caused primarily by Akabane virus (AKAV), characterized by abortion, premature birth, stillbirth, congenital joint contractures, and arthrogryposis-hydranencephaly syndrome (AH) in cattle and sheep (1, 2). Additionally, AKAV can infect avian embryos, mice, and hamsters, leading to death. AKAV is widespread in Africa, the Middle East, East Asia, Southeast Asia, and Australia (3–5). Between 1972 and 1975, a serious outbreak of Akabane disease broke out in Japan, with the original strain JaGAr39 isolated from mosquitoes in Japan in 1959, resulting in over 31,000 cases of abortion, stillbirth, congenital joint diseases, and AH syndrome cases (6). In 1974, AKAV-induced AH syndrome was prevalent in Australia, when more than 8,000 confirmed cases were reported (7). In 1998, AKAV was first isolated from mosquitoes in Shanghai, China, and named the SH-1 strain (8). The global prevalence of AKAD exhibits distinct regional, periodic, and seasonal patterns (9) and can spread across species, causing significant economic losses to livestock industries in endemic regions and posing a long-term threat to animal health and sustainable farming practices. As the envelope glycoprotein of AKAV, Gc not only serves as a crucial target for the immune system of vertebrate hosts, eliciting strong specific immune responses, but also plays a decisive role in various important biological properties of AKAV, such as pathogenicity, neutralizing activity, hemagglutination ability (10–12). It is crucial to study the key aspects of the AKAV Gc gene to improve the economic viability of livestock farming and ensure its stable development.

## 2 Materials and methods

### 2.1 The dataset

From the GenBank database on the NCBI website, a total of 147 AKAV Gc strains sequences were selected, comprising 61 strains from Japan, 39 from the United States, 35 from China, 2 from Israel, 9 from South Korea, and 1 from Turkey, spanning lineages I-V (see Supplementary Table 1). These strains encompass different years from 1959 to 2023, including the majority of AKAV strains from China. The genetic variation of the AKAV Gc gene sequence over the past 64 years has helped to analyze the evolution of AKAV Gc and provide a theoretical basis for AKAV prevention and control. Out of the 147 AKAV strains, 45 strains were carefully selected for further Gc sequence analysis, representing strains from lineages I-V, including reference strains, vaccine strains, and epidemic strains, to ensure a comprehensive analysis of genetic variations in the AKAV Gc gene (Table 1).

**Table 1:** Information of 45 selected AKAV strains.

**Number**	**Strain**	**Area**	**Year**	**GenBank**
1	JaLAB39	Australia	1959	KR260715
2	JaGAr39	Japan	1959	AB297818
3	B8935	Australia	1968	AB297848
4	R7949	Japan	1968	AB297849
5	MP496	Kenya	1972	AB297850
6	CS0016	Australia	1975	MH734998
7	DPP231	Australia	1977	MH735015
8	KT3377	Japan	1977	AB297819
9	CS661	Australia	1980	MH735026
10	C1316	Australia	1980	MH735028
11	CS979	Australia	1981	MH735027
12	CS231	Australia	1982	MH735019
13	KS-1/E/85	Japan	1985	AB297821
14	KSB-1/C/87	Japan	1987	AB297822
15	ON-3/E/90	Japan	1990	AB297832
16	FO-90-3	Japan	1990	AB297831
17	93H78	China	1993	MF278864
18	K9	South Korea	1993	FJ498798
19	ON-5/B/98	Japan	1998	AB297842
20	ON-1/E/98	Japan	1998	AB297841
21	CB-1/F/98	Japan	1998	AB297843
22	Okayama2001	Japan	2001	AB289322
23	Okayama2004	Japan	2004	AB289323
24	K0505	South Korea	2005	FJ498800
25	OBE-1	Japan	2006	NC_009895
26	OS-1/Pl/08	Japan	2008	LC217491
27	NG-1/P/08	Japan	2008	LC217492
28	FI-1/Br/08	Japan	2008	LC552051
29	HN10169	China	2010	MG731555
30	MZ-1/Br/11	Japan	2011	LC217502
31	EH-3/Br/11	Japan	2011	LC217501
32	PT-1/AKA/C/12/TW	China	2012	MF278857
33	12H37	China	2012	MF278861
34	52	China	2013	OR387106
35	55	China	2013	OR387107
36	CX-01	China	2015	MW194115
37	NM/BS/1	China	2016	KU375443
38	GXDH01	China	2016	MH174978
39	GXLCH70N	China	2016	KY381281
40	TJ2016	China	2016	MT761688
41	ISR-256/16	Israel	2016	MZ547651
42	ISR-170/18	Israel	2018	MW822047
43	Iriki	Japan	2018	AB289324
44	CH-JL-01-2022	China	2022	PQ560879
45	AKAV-7	South Korea	2023	PQ799180

### 2.2 Analysis of Akabane virus gene sequences

The nucleotide similarity of 45 strains selected from 147 strains of AKAV Gc was analyzed. These 45 AKAV strains cover classical strains and vaccine strains in different countries and different periods. The nucleotide homology of Gc gene was analyzed from NCBI website (https://www.ncbi.nlm.nih.gov/) by Clustal W method in MegAlign function of DNAStar software (version 7.0, Madison, WI). The phylogenetic analysis of 147 AKAV Gc sequences (Supplementary Table 1) was carried out by using the maximum likelihood method (ML) with 1,000 Bootstrap repeated sampling of MEGA software (7th edition, Mega Limited, Auckland, New Zealand). Subsequently, these sequences were annotated with a network tool called interactive tree of life.

## 3 Descriptive results

**Figure 1:**
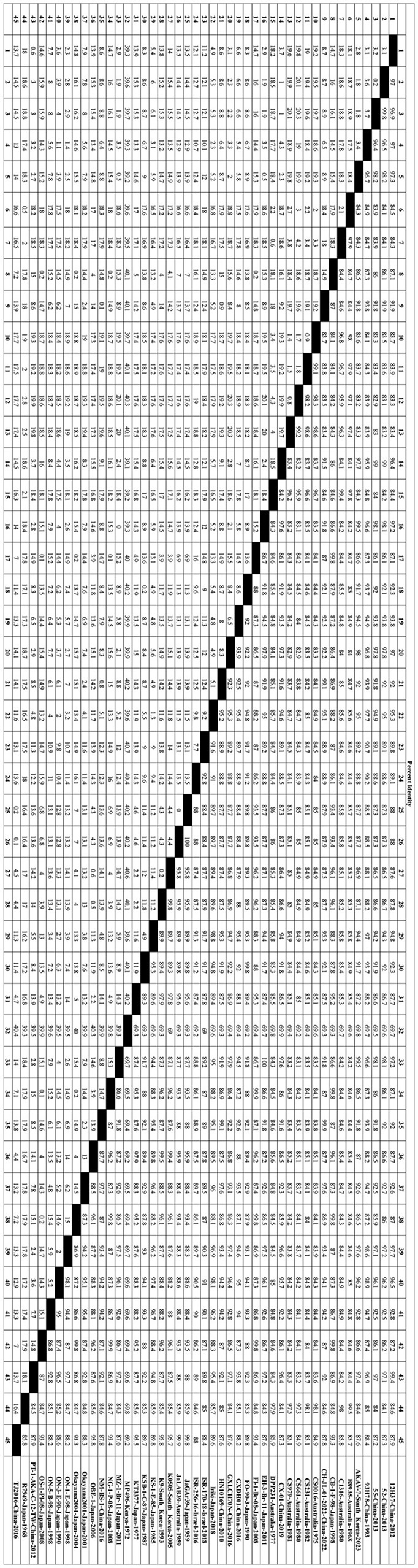
Forty-five representative AKAV strains (Table 1) were selected to obtain Gc individual sequences for nucleotide homology analysis. The nucleotide homology of Gc sequences was analyzed using the Clustal W algorithm in DNAStar software (version 7.0, Madison, WI).

**Figure 2:**
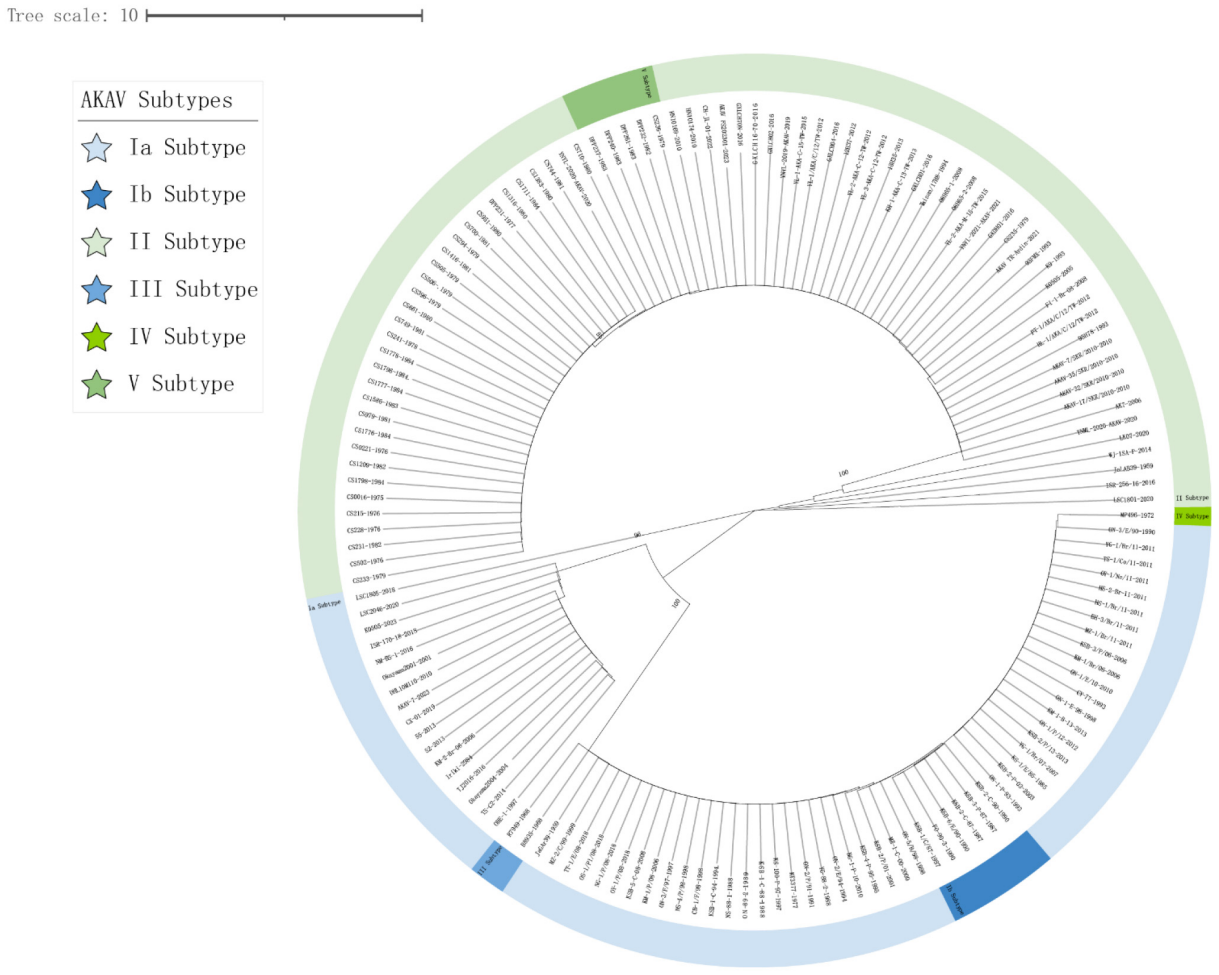
Phylogenetic analysis of AKAV Gc genes (Supplementary Table 1). Clustal W alignment was conducted using the MegAlign feature in DNAStar software (version 7.0). Subsequently, the maximum likelihood method with 1,000 bootstrap replicates was performed using MEGA software (version 11.0). The generated trees were visualized and annotated using the online “The Interactive Tree of Life” (https://itol.embl.de, accessed on 20 May 2025) software. The five subtypes (I–V) of AKAV, Ia, Ib, II, III, IV, and V, are labeled.

In this study, 45 representative isolates were systematically selected from the initial 147 strains. These isolates covered a wide range of time (1959–2023) and geography, including Japan, China, South Korea, Australia, and Israel. They also represented five known genetic families (I to V). Key reference strains like the prototype strain JaGAr39, vaccine strain OBE-1, and genetically distinct isolates such as MP496 were included for a comprehensive analysis. The phylogenetic tree, based on Gc protein sequences, displayed significant genetic diversity. Amino acid homology similarity ranged from 69.2% to 100% (refer to Figure 1). The maximum likelihood phylogenetic tree was utilized to analyze the relationship between these strains further, highlighting conservation and variation (refer to Figure 2).

Notably, the Kenyan strain MP496 formed a unique and deeply branched pedigree (pedigree IV), showing stark differences from all isolates in Southeast Asia and Australia. Its nucleotide homology with other AKAV isolates ranged from 69.2% to 69.8%, with the highest similarity to R7949 at 69.8% and the lowest with OBE-1 and TJ2016 at 69.2%.

In contrast, pedigree I included strains like CY-77 from Taiwan Province, Iriki, and ON-1/E/98 from Japan, while pedigree II encompassed vaccine strains OBE-1, JaGAr39, and KT3377, forming a major Asian branch. Australian isolates B8935 and R7949 clustered together into lineage III, underlining the impact of geographical isolation on AKAV evolution. Isolates from the same country and year displayed high nucleotide homology (>99%). For instance, Chinese isolates 52 and 55 exhibited a 99.8% homology, while Japanese isolates JaGAr39 and JaLAB39 showed 100% similarity, suggesting a shared ancestral origin. Australian isolates B8935, CS0016, and CS231, collected from 1968 to 1982, formed a closely related phylogenetic group with 97.1%-99.5% nucleotide homology. The Korean strain AKAV-7 shared 83.1%-85.7% nucleotide homology with the Chinese strain PT-1-AKA-C-12-TW.

Between 2010 and 2023, East Asian isolates from China, South Korea, and Japan formed a highly homologous group, with nucleotide homology exceeding 97%. For example, the Korean strain AKAV-7 had a 99.5% homology with Japanese strains EH-3-Br-11 and MZ-1-Br-11, indicating recent cross-border transmission of closely related virus populations. These results underscore the combined impacts of geographical isolation and time evolution on AKAV genetic diversity.

The comprehensive analysis of similarity and diversity among these AKAV strains has unveiled the genetic evolution of the AKAV Gc gene across different regions and time points. By comparing the amino acid sequences of these strains, a better understanding of the population structure and transmission dynamics of AKAV is gained. High similarity between specific strains may suggest shared transmission pathways within the same region or time period, while lower similarity could indicate genetic differentiation and evolution across different regions or time points. Furthermore, through phylogenetic analysis, the phylogenetic relationships and classification among different subtypes of AKAV can be further elucidated. This aids in comprehending the geographical distribution and pathogenic differences among various subtypes, providing crucial insights for tailored disease control strategies. The associations between different subtypes or strains may be linked to various factors such as host population structure, environmental influences, and viral transmission pathways. Combining the results of amino acid similarity and phylogenetic analysis allows for a more comprehensive understanding of the genetic diversity and transmission pathways of AKAV. This helps predict the transmission potential of different subtypes or strains, assess the risk of virus epidemics, and provide guidance for future disease surveillance and control measures. Through in-depth exploration of genetic variations and evolutionary mechanisms of the AKAV Gc gene, better preparation can be made for potential epidemic threats, safeguarding the health and sustainable development of the livestock industry. These research findings provide crucial scientific foundations for understanding the transmission, pathogenesis, and epidemiology of AKAV, offering robust support for future disease prevention and control efforts.

## Data Availability

The datasets presented in this study can be found in online repositories. The names of the repository/repositories and accession number(s) can be found in the article/Supplementary material.

## References

[1] De Regge N. Akabane, Aino and Schmallenberg virus-where do we stand and what do we know about the role of domestic ruminant hosts and Culicoides vectors in virus transmission and overwintering? Curr Opin Virol. (2017) 27:15–30. doi: 10.1016/j.coviro.2017.10.004 29096232

[2] Qiao J Chen C Ren Y. Research progress on Akabane disease. J Tarim Univ. (2008) 103–6. doi: 10.1063/1.2837055

[3] Taylor WP Mellor PS. The distribution of Akabane virus in the Middle East. Epidemiol Infect. (1994) 113:175–85. doi: 10.1017/S0950268800051591 8062874 PMC2271212

[4] Jun Q Qingling M Zaichao Z Kuojun C Jingsheng Z Minxing M . serological survey of akabane virus infection in cattle and sheep in northwest China. Trop Anim Health Prod. (2012) 44:1817–20. doi: 10.1007/s11250-012-0168-3 22581316

[5] Kono R Hirata M Kaji M Goto Y Ikeda S Yanase T . Bovine epizootic encephalomyelitis caused by Akabane virus in southern Japan. BMC Vet Res. (2008) 4:20. doi: 10.1186/1746-6148-4-20 18554406 PMC2443122

[6] Brenner J Yanase T Kato T Yaakobi S Khinich E Paz R . Serological evidence suggests that several Simbu serogroup viruses circulated in Israel. Vet Ital. (2019) 55:81–9. doi: 10.12834/VetIt.1397.7622.2 30951185

[7] Coverdale OR Cybinski DH St George TD. Congenital abnormalities in calves associated with Akabane virus and Aino virus. Aust Vet J. (1978) 54:151–2. doi: 10.1111/j.1751-0813.1978.tb05538.x 687271

[8] Yadav P Shete A Bondre V Patil D Kokate P Chaudhari S . Isolation and characterization of Oya virus a member of Simbu serogroup, family Bunyaviridae, isolated from Karnataka, India. Infect Genet Evol: J Mol Epidemiol Evol Genet Infect Dis. (2016) 44:122–6. doi: 10.1016/j.meegid.2016.06.049 27374486

[9] Li C Xue F Wang K Wang Y Ma X. Research progress on the epidemiology and prevention of Akabane disease. Adv Vet Med. (2023) 44:96–100. doi: 10.16437/j.cnki.1007-5038.2023.06.022

[10] Yanase T Yoshida K Ohashi S Kato T Tsuda T. Sequence analysis of the medium RNA segment of three Simbu serogroup viruses, Akabane, Aino, and Peaton viruses. Virus Res. (2003) 93:63–9. doi: 10.1016/S0168-1702(03)00066-2 12727343

[11] Xu S Tong T Bai Y Wang Q Zhang W Sun Q . Eukaryotic expression and antigenicity detection of Akabane virus G1 gene. Chin J Prev Vet Med. (2008) 440–4.

[12] Lan X Liang F Li G Kong W Wang R Wang L . Research progress on the GC proteins of Akabane virus. Vet Sci. (2025) 12:701. doi: 10.3390/vetsci12080701 40872652 PMC12390303

