# Supplementary material for: Genetic evolution analysis of AKAV Gc gene
Source: Front Vet Sci. 2025 Nov 13;12:1715626. doi: 10.3389/fvets.2025.1715626 (PMC12659180; doi:10.3389/fvets.2025.1715626)
Supplement: Supplementary file 1 [file Table_1.docx]

| **Table S1. The AKAV Gc Segment reference sequences.** | | | | |
| --- | --- | --- | --- | --- |
| Year | Area | Strain | Genbank Accession number |  |
| 1959 | Japan | JaGAr39 | AB297818 |  |
| 1959 | Australia | JaLAB39 | KR260715 |  |
| 1968 | Australia | B8935 | AB297848 |  |
| 1968 | Japan | R7949 | AB297849 |  |
| 1972 | Japan | MP496 | AB297850 |  |
| 1975 | Australia | CS0016 | MH734998 |  |
| 1976 | Australia | CS0221 | MH734999 |  |
| 1976 | Australia | CS215 | MH735017 |  |
| 1976 | Australia | CS228 | MH735018 |  |
| 1976 | Australia | CS502 | MH735024 |  |
| 1977 | Japan | KT3377 | AB297819 |  |
| 1977 | Australia | DPP231 | MH735015 |  |
| 1978 | Australia | CS241 | MH735023 |  |
| 1979 | Australia | CS294 | MH735000 |  |
| 1979 | Australia | CS396 | MH735001 |  |
| 1979 | Australia | CS505 | MH735002 |  |
| 1979 | Australia | CS233 | MH735020 |  |
| 1979 | Australia | CS235 | MH735021 |  |
| 1979 | Australia | CS236 | MH735022 |  |
| 1979 | Australia | CS506 | MH735025 |  |
| 1980 | Australia | CS661 | MH735026 |  |
| 1980 | Australia | CS1383 | MH735029 |  |
| 1980 | Australia | CS710 | MH735004 |  |
| 1980 | Australia | CS951 | MH735007 |  |
| 1980 | Australia | C1316 | MH735028 |  |
| 1981 | Australia | CS700 | MH735003 |  |
| 1981 | Australia | CS744 | MH735005 |  |
| 1981 | Australia | CS749 | MH735006 |  |
| 1981 | Australia | CS1416 | MH735009 |  |
| 1981 | Australia | CS979 | MH735027 |  |
| 1982 | Australia | CS1209 | MH735008 |  |
| 1982 | Australia | CS231 | MH735019 |  |
| 1982 | Australia | DPP232 | MH735032 |  |
| 1983 | Australia | CS1586 | MH735030 |  |
| 1983 | Australia | DPP237 | MH735033 |  |
| 1983 | Australia | DPP240 | MH735034 |  |
| 1983 | Australia | DPP261 | MH735035 |  |
| 1984 | Japan | Iriki | AB289324 |  |
| 1984 | Australia | CS1711 | MH735010 |  |
| 1984 | Australia | CS1776 | MH735011 |  |
| 1984 | Australia | CS1777 | MH735012 |  |
| 1984 | Australia | CS1778 | MH735013 |  |
| 1984 | Australia | CS1798 | MH735014 |  |
| 1984 | Australia | CS1796 | MH735031 |  |
| 1985 | Japan | KS-1/E/85 | AB297821 |  |
| 1987 | Japan | KSB-1/C/87 | AB297822 |  |
| 1987 | Japan | KSB-1/C/87 | AB297822 |  |
| 1987 | Japan | KSB-2/C/87 | AB297823 |  |
| 1987 | Japan | KSB-3/P/87 | AB297824 |  |
| 1988 | Japan | KSB-1/C/88 | AB297826 |  |
| 1988 | Japan | NS-88-1 | AB297825 |  |
| 1988 | Japan | YG-88-2 | AB297827 |  |
| 1989 | Japan | ON-89-2 | AB297828 |  |
| 1990 | Japan | KSB-2/C/90 | AB297829 |  |
| 1990 | Japan | KSB-6/E/90 | AB297830 |  |
| 1990 | Japan | FO-90-3 | AB297831 |  |
| 1990 | Japan | ON-3/E/90 | AB297832 |  |
| 1991 | Japan | ON-2/P/91 | AB297833 |  |
| 1993 | Japan | ON-1/P/93 | AB297834 |  |
| 1993 | Japan | CY-77 | AB297851 |  |
| 1993 | South Korea | K9 | FJ498798 |  |
| 1993 | South Korea | 93FMX | FJ498799 |  |
| 1993 | China | 93H78 | MF278864 |  |
| 1994 | Japan | KSB-1/C/94 | AB297836 |  |
| 1994 | Japan | ON-2/E/94 | AB297835 |  |
| 1994 | China | Tainan/17H8 | MF278865 |  |
| 1995 | Japan | KSB-4/P/95 | AB297837 |  |
| 1997 | Japan | KS-100/P/97 | AB297838 |  |
| 1997 | Japan | ON-3/F/97 | AB297839 |  |
| 1997 | Japan | OBE-1 | NC_009895 |  |
| 1998 | Japan | NS-4/P/98 | AB297840 |  |
| 1998 | Japan | ON-1/E/98 | AB297841 |  |
| 1998 | Japan | ON-5/B/98 | AB297842 |  |
| 1998 | Japan | CB-1/F/98 | AB297843 |  |
| 1999 | Japan | MZ-2/C/99 | AB297844 |  |
| 2000 | Japan | MZ-1/C/00 | AB297845 |  |
| 2001 | Japan | Okayama2001 | AB289322 |  |
| 2001 | Japan | KSB-2/P/01 | AB297846 |  |
| 2003 | Japan | KSB-2/P/03 | AB297847 |  |
| 2004 | Japan | Okayama2004 | AB289323 |  |
| 2005 | South Korea | K0505 | FJ498800 |  |
| 2006 | South Korea | AK7 | FJ498801 |  |
| 2006 | Japan | KM-2/Br/06 | AB426281 |  |
| 2006 | Japan | KSB-3/P/06 | AB426282 |  |
| 2006 | Japan | KM-1/Br/06 | AB436954 |  |
| 2007 | Japan | YG-1/Br/07 | LC217488 |  |
| 2008 | Japan | KSB-5/C/08 | AB568603 |  |
| 2008 | Japan | TT-1/E/08 | LC217489 |  |
| 2008 | Japan | OY-1/P/08 | LC217490 |  |
| 2008 | Japan | OS-1/Pl/08 | LC217491 |  |
| 2008 | Japan | NG-1/P/08 | LC217492 |  |
| 2008 | Japan | KM-1/P/08 | LC217493 |  |
| 2008 | China | 08H65-1 | MF278862 |  |
| 2008 | China | 08H65-2 | MF278863 |  |
| 2010 | South Korea | AKAV-7/SKR/2010 | JQ308775 |  |
| 2010 | South Korea | AKAV-17/SKR/2010 | JQ308776 |  |
| 2010 | South Korea | AKAV-32/SKR/2010 | JQ308777 |  |
| 2010 | South Korea | AKAV-35/SKR/2010 | JQ308778 |  |
| 2010 | China | DHL10M110 | KY284022 |  |
| 2010 | Japan | ON-1/E/10 | LC217494 |  |
| 2010 | China | HN10169 | MG731555 |  |
| 2010 | China | HN10174 | MG731556 |  |
| 2011 | Japan | OY-1/Ne/11 | LC217496 |  |
| 2011 | Japan | YG-1/Br/11 | LC217499 |  |
| 2011 | Japan | TS-1/Ce/11 | LC217500 |  |
| 2011 | Japan | MZ-1/Br/11 | LC217502 |  |
| 2012 | China | YL-3/AKA/C/12/TW | MF278859 |  |
| 2012 | Japan | ON-1/P/12 | LC217503 |  |
| 2012 | China | YL-1/AKA/C/12/TW | MF278855 |  |
| 2012 | China | YL-2/AKA/C/12/TW | MF278856 |  |
| 2012 | China | PT-1/AKA/C/12/TW | MF278857 |  |
| 2012 | China | HL-1/AKA/C/12/TW | MF278858 |  |
| 2012 | China | 12H37 | MF278861 |  |
| 2013 | Japan | KM-1/B/13 | LC217504 |  |
| 2013 | Japan | KSB-2/P/13 | LC217505 |  |
| 2013 | China | 13H35 | MF278860 |  |
| 2013 | China | KH-1/AKA/C/13/TW | MT676825 |  |
| 2013 | China | 52 | OR387106 |  |
| 2013 | China | 55 | OR387107 |  |
| 2014 | Japan | TS-C2 | AB968526 |  |
| 2014 | Japan | HS-1/Br/11 | LC217497 |  |
| 2015 | China | YL-1/AKA/C/15/TW | MT676826 |  |
| 2015 | China | YL-2/AKA/M/15/TW | MT676827 |  |
| 2016 | China | NM/BS/1 | KU375443 |  |
| 2016 | China | GXLCH01 | KY381277 |  |
| 2016 | China | GXLCH02 | KY381278 |  |
| 2016 | China | GXLCH04 | KY381279 |  |
| 2016 | China | GXLCH16-70 | KY381280 |  |
| 2016 | China | GXLCH70N | KY381281 |  |
| 2016 | Japan | HS-2/Br/11 | LC217498 |  |
| 2016 | Japan | EH-3/Br/11 | LC217501 |  |
| 2016 | Japan | FI-1/Br/08 | LC552051 |  |
| 2016 | China | GXDH01 | MH174978 |  |
| 2016 | China | TJ2016 | MT761688 |  |
| 2016 | Israel | ISR-256/16 | MZ547651 |  |
| 2018 | Israel | ISR-170/18 | MW822047 |  |
| 2019 | China | CX-01 | MW194115 |  |
| 2019 | China | YNYL-2019-AKAV | PP918938 |  |
| 2020 | Japan | HG-1/P/10 | LC217495 |  |
| 2020 | China | LK07 | OP432313 |  |
| 2020 | China | YNYL-2020-AKAV | PP918939 |  |
| 2020 | China | YNML-2020-AKAV | PP918941 |  |
| 2021 | Turkey | AKAV_TR/Aydin/2021 | PP785401 |  |
| 2021 | China | YNYL-2021-AKAV | PP918940 |  |
| 2022 | China | CH-JL-01-2022 | PQ560879 |  |
| 2023 | China | AKAV_FS202301 | PQ567127 |  |
| 2023 | South Korea | AKAV-7 | PQ799180 |  |
